# Maternal Western-Style High Fat Diet Induces Sex-Specific Physiological and Molecular Changes in Two-Week-Old Mouse Offspring

**DOI:** 10.1371/journal.pone.0078623

**Published:** 2013-11-05

**Authors:** Mona Mischke, Maurien G. M. Pruis, Mark V. Boekschoten, Albert K. Groen, Aditia R. Fitri, Bert J. M. van de Heijning, Henkjan J. Verkade, Michael Müller, Torsten Plösch, Wilma T. Steegenga

**Affiliations:** 1 Nutrition, Metabolism & Genomics Group, Division of Human Nutrition, Wageningen University, Wageningen, The Netherlands; 2 Center for Liver, Digestive and Metabolic Diseases, Department of Pediatrics, University Medical Center Groningen, University of Groningen, Groningen, The Netherlands; 3 Department for Laboratory Medicine, University Medical Center Groningen, University of Groningen, Groningen, The Netherlands; 4 Danone Research, Centre for Specialised Nutrition, Wageningen, The Netherlands; INRA, France

## Abstract

Maternal diet is associated with the development of metabolism-related and other non-communicable diseases in offspring. Underlying mechanisms, functional profiles, and molecular markers are only starting to be revealed. Here, we explored the physiological and molecular impact of maternal Western-style diet on the liver of male and female offspring. C57BL/6 dams were exposed to either a low fat/low cholesterol diet (LFD) or a Western-style high fat/high cholesterol diet (WSD) for six weeks before mating, as well as during gestation and lactation. Dams and offspring were sacrificed at postnatal day 14, and body, liver, and blood parameters were assessed. The impact of maternal WSD on the pups’ liver gene expression was characterised by whole-transcriptome microarray analysis. Exclusively male offspring had significantly higher body weight upon maternal WSD. In offspring of both sexes of WSD dams, liver and blood parameters, as well as hepatic gene expression profiles were changed. In total, 686 and 604 genes were differentially expressed in liver (*p*≤0.01) of males and females, respectively. Only 10% of these significantly changed genes overlapped in both sexes. In males, in particular alterations of gene expression with respect to developmental functions and processes were observed, such as Wnt/beta-catenin signalling. In females, mainly genes important for lipid metabolism, including cholesterol synthesis, were changed. We conclude that maternal WSD affects physiological parameters and induces substantial changes in the molecular profile of the liver in two-week-old pups. Remarkably, the observed biological responses of the offspring reveal pronounced sex-specificity.

## Introduction

Incidence of metabolic syndrome and other non-communicable diseases increased epidemic-like over the last decades. This not only puts a heavy burden on the individual, but also on the health care systems worldwide [1], [2]. It is commonly accepted that food patterns, such as a Western-style high fat diet (WSD), have a causal link to the development of metabolic syndrome and cardiovascular diseases [3]–[5]. In general, WSDs refer to highly processed diets that are rich in calories and have a high (saturated) fat and cholesterol content. Nowadays, this kind of food pattern is increasingly replacing a healthy low fat diet (LFD) in Western, as well as in newly industrialized countries (‘emerging economies’) [6]–[9].

Apart from the direct impact of nutrition on the individual’s health, an increasing number of studies substantiates that early-life nutritional cues co-determine the health status in later life by metabolic programming [10]–[13]. Currently, understanding of the metabolic programming mechanisms is still incomplete. Yet, evidence from human and animal studies progressively demonstrates that early-life nutrition affects the offspring’s epigenome, which is associated with gene expression status and adult health eventually [14]–[17]. Besides the pre-conceptional period, where the oocyte and sperm can be affected by nutritional and environmental factors [18], [19], the foetal and early post-natal stages seem to be the main susceptible periods for metabolic programming [13], [20]. In these perinatal periods, offspring’s nourishment is essentially provided by the mother, therefore basically linked to the maternal diet.

To date, the impact of varying maternal high fat diets (HFD) on the adult offspring was investigated in several animal studies, reporting primarily on physiological outcomes. Essentially, features of metabolism-related diseases were observed, including obesity, hypertension, poor glycaemic control, dyslipidaemia, and the development of diabetes and fatty liver [21]–[27]. Thereby, sex-specificity regarding nature and severity of effects was reported [21]–[25], [27], [28]. Knowledge of related molecular mechanisms is scarce and results mainly from targeted approaches. Hence, it is fragmentary and limited to selective genes and functions, including alterations in the hypothalamic regulation of energy homeostasis, as well as regulation of a limited number of genes and proteins related to liver development and function [14], [23], [25], [27], [29]–[31].

Moreover, most studies on the effects of maternal diet are designed to determine long-term effects on the offspring’s health, and focus on the adult period when disease patterns are distinct. During growing up, numerous factors act upon the offspring, which presumably blur the critical molecular impact of the maternal diet that account for metabolic programming effects. To circumvent this problem and to eliminate influencing factors apart from maternal diet, analysis of pre-weaning offspring will elucidate the direct molecular impact of maternal diet. This might contribute to the understanding of the influence of maternal environment, such as diet, on the offspring’s health status in adult life.

In the present study, we aimed to characterise the direct molecular impact of maternal WSD and its relation to the physiology of young pre-weaning C57BL/6 offspring. Importantly, we also considered potential sex-specificity of effects by analysing both male and female two-week-old offspring of dams receiving either a LFD or a WSD. In all mice, body, liver, and blood lipid parameters were determined and the liver transcriptome was monitored by microarray analysis. Our results indicate that maternal WSD has a clear impact on young offspring, which shows a strong sex-dependency for the physiological, as well as for the molecular outcome.

## Methods

### Ethics Statement

The national and institutional guidelines for the care and use of animals were followed, and the experimental procedures were reviewed and approved by the Ethics Committees for Animal Experiments of the University of Groningen, The Netherlands (ethics registration code 5709). All efforts were made to minimize suffering.

### Animals and Diets

**Figure 1:**
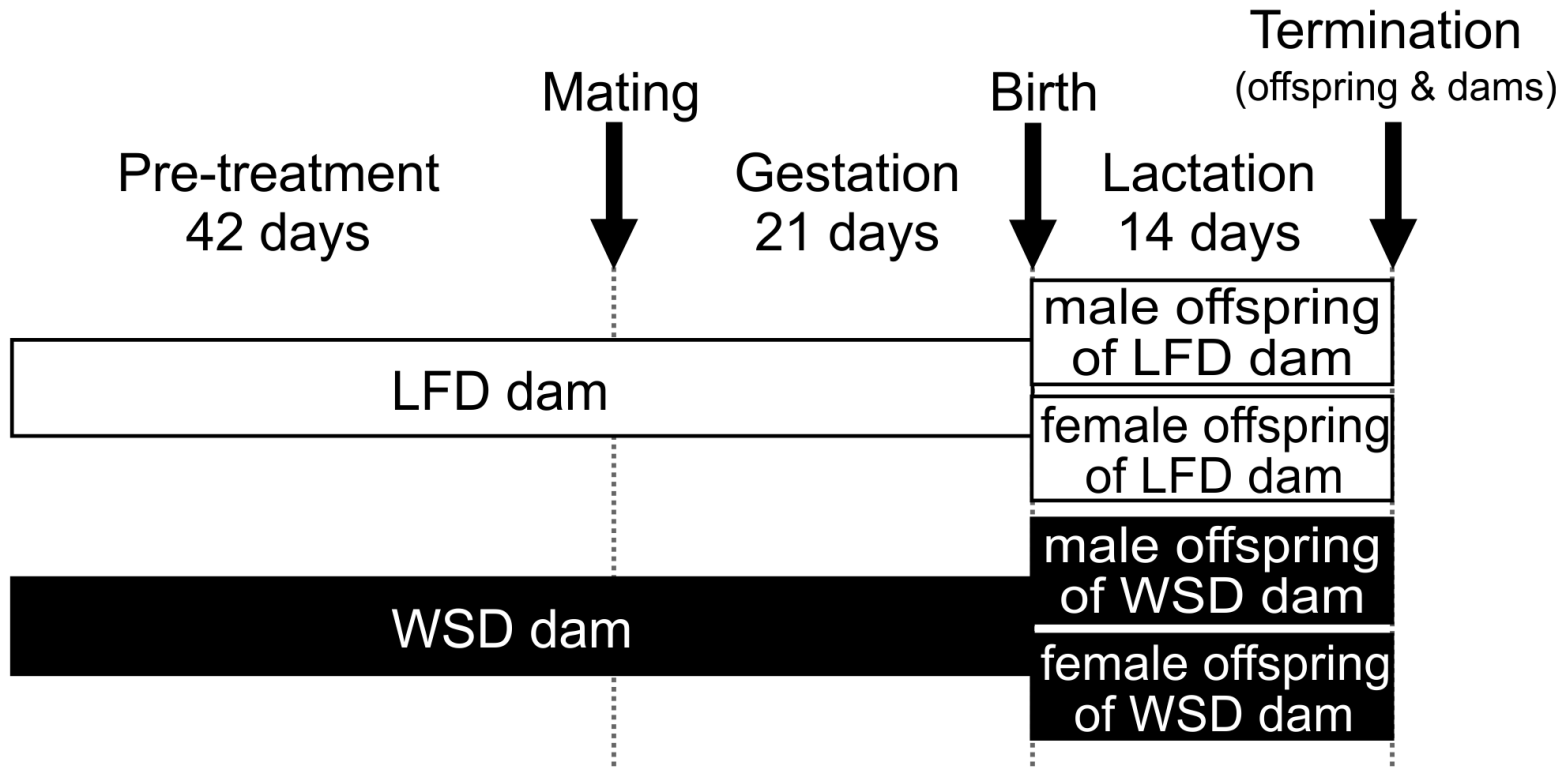
Study design. C57BL/6 dams received either a low fat control diet (LFD) or a Western-style high fat diet (WSD) throughout the study. The treatment started six weeks before mating and continued during gestation and lactation. Dams and offspring were sacrificed at postnatal week two. Male and female offspring were included into further analysis: male offspring from maternal LFD (n = 6), male offspring from maternal WSD (n = 6), female offspring from maternal LFD (n = 9), female offspring from maternal WSD (n = 6).

Female C57BL/6 mice (five weeks of age) were purchased from Harlan (Horst, The Netherlands) and housed individually in the light- and temperature-controlled facility of the University Medical Center Groningen (lights on 7:00 am–7:00 pm, 21°C). The mice had free access to drinking water and were randomly assigned to either a semi-synthetic low fat control diet (LFD, 3.85 kcal/g; 10 E% fat, 20 E% protein, 70 E% carbohydrate; D12450B, Research Diets, New Brunswick, USA) that contained low amounts of cholesterol from lard (18.0 mg cholesterol/kg) or a semi-synthetic energy rich Western-style high fat diet (WSD, 4.73 kcal/g; 45 E% fat, 20 E% protein, 35 E% carbohydrate; D12451, Research Diets) that contained a high cholesterol content from lard (196.5 mg cholesterol/kg). After 6 weeks on their respective diets (pre-treatment period), the female mice were mated with males on control diet. In case conceiving failed, mice were allowed to re-mate. Throughout pregnancy and lactation, the dams received the same diets as during pre-treatment (Figure 1). Mice were allowed to deliver spontaneously and were left undisturbed with their litters for 24 h. Litter sizes were standardized to 5–7 pups, to ensure no litter was nutritionally biased. By natural circumstances, the litter size of some dams was reduced further, but not changing the overall male/female ratio within the diet significantly. Two weeks into lactation, dams and offspring were sacrificed by cervical dislocation under isoflurane anaesthesia. Blood was collected by cardiac puncture and livers were dissected, weighed, snap-frozen in liquid nitrogen, and stored at −80°C until further use. Both male and female offspring were included into further analysis, resulting in four experimental groups: male offspring from maternal LFD (m-LF; n = 6), male offspring from maternal WSD (m-WS; n = 6), female offspring from maternal LFD (f-LF; n = 9), female offspring from maternal WSD (f-WS; n = 6).

### Plasma lipids

Commercially available kits were used according to the manufacturers’ instructions to determine the lipid profiles, including triglycerides (Roche Diagnostics, Mannheim, Germany), total and non-esterified cholesterol (DiaSys Diagnostic Systems, Holzheim, Germany), and non-esterified fatty acids (Wako, Neuss, Germany) in blood plasma of dams and offspring.

### RNA Isolation

Total RNA was isolated from liver samples using TRIzol reagent (Invitrogen, Breda, The Netherlands), treated with DNAse, and purified on columns (RNAeasy microkit, Qiagen, Venlo, The Netherlands), all according to the manufacturers’ instructions. Purified RNA was immediately stored at −80°C until further use. RNA concentrations were determined using the NanoDrop ND-1000 UV-vis spectrophotometer (Isogen, Maarsen, The Netherlands). RNA integrity was verified on an Agilent 2100 Bioanalyzer with the 6000 Nano Kit using the Eukaryote Total RNA Nano assay according to the manufacturer’s instructions (Agilent Technologies, Amsterdam, The Netherlands). Samples were considered suitable for hybridization when they showed intact bands of 18S and 28S ribosomal RNA subunits, displayed no chromosomal peaks or RNA degradation products, and had a RNA integrity number (RIN) above 8.0.

### Microarray Hybridization and Analysis

Per offspring liver sample, 100 ng of purified RNA was used for the preparation of labelled cDNA, applying the Ambion Whole Transcript (WT) Expression kit (Life Technologies, Carlsbad, USA) in combination with the Affymetrix GeneChip WT Terminal Labelling kit (Affymetrix, Santa Clara, USA). All samples were hybridized at one time point to Affymetrix GeneChip Mouse Gene 1.1 ST arrays according to standard Affymetrix protocols. Quality control and normalisation were performed using Bioconductor software packages integrated in an on-line pipeline [32]. Normalised expression estimates of probe sets were computed by the robust multiarray (RMA) analysis algorithm available in the Bioconductor library AffyPLM using default settings [33]. Probe sets were redefined according to Dai *et al.* [34] and assigned to unique gene identifiers (IDs) of the Entrez Gene database, resulting in 21,225 assigned Entrez IDs. Array data were submitted to the Gene Expression Omnibus and are available under accession number GSE46359.

### Bioinformatic Analysis

Of the 21,225 defined genes covered by the microarray, only genes with an intensity value of ≥20 on at least 4 arrays and an interquartile range (IQR)≥0.1 were selected for further analysis. The remaining 11,362 genes were ranked based on their IQR value, and principal component analysis (PCA) was applied on the top 1,000 most variable genes using MultiExperimentViewer, version 4.8.1 [35], [36]. Signal 2log ratios, which represent fold changes (FC), and related significances of change were calculated from the mean signal intensities of maternal WSD groups and maternal LFD groups using intensity based-moderated t-statistics (IBMT) implementing empirical Bayes correction [37]. Resulting 2log ratios and *p*-values were applied for further descriptive bioinformatic analysis of the data. The method description and results of quantitative real-time PCRs that confirm the microarray data are presented as Supporting Information (Method S1 and Figure S1).

Ingenuity Pathway Analysis (IPA, Ingenuity^®^ Systems, www.ingenuity.com) was used to relate the microarray data to biological functions and canonical pathways. Genes that significantly changed in expression due to maternal diet (*p*-value≤0.01) were subjected to this comprehensive pathway and network analysis.

Gene sets related to biological functions that were most significant to the data, were expressed in heat maps using MultiExperimentViewer, version 4.8.1 [35], [36]. By hierarchical clustering based on Pearson correlation, gene data were organized into binary trees that group similar elements together.

### Statistical analysis

All physiological data are expressed as means ± standard deviation (SD). In dams, the differences between the mean values of the two diet groups were tested for statistical significance with a Student’s t-test. In offspring, effects of maternal WSD were tested by comparing mean values within one sex-group using one-way ANOVA and the Tukey post hoc test (SPSS Inc., Chicago, USA). For all tests, *p*-values≤0.05 were considered statistically significant.

## Results

### Body, liver, and blood parameters of dams and two-week-old offspring

To determine the impact of maternal diet on the body’s physiology and molecular function of the offspring’s liver, C57BL/6 dams were fed either a low fat diet (LFD, 10 E% fat, 18.0 mg cholesterol/kg) or Western-style high fat diet (WSD, 45 E% fat, 196.5 mg cholesterol/kg) six weeks pre-mating and continued during pregnancy and lactation (Figure 1). Dams on LFD and WSD had similar body weights at the different measuring time points (data not shown), except for the sacrifice time point. At sacrifice, WSD dams had a significantly higher body weight than LFD dams, accompanied by lower liver weight/body weight ratios at similar liver weights (Table 1). Fasted blood parameters, including triglycerides (TG), cholesterol, and non-esterified fatty acids (NEFA) were comparable at this time point.

**Table 1:** Physiological parameters in dams fed LFD or WSD at two weeks post-delivery.

	Low fat	Western-style
Body weight (g)	27.35±1.67	32.21±1.19*
Liver weight (g)	2.28±0.31	2.00±0.15
Ratio liver/body weight (%)	8.33±0.64	6.22±0.31**
**Plasma lipids**		
Triglycerides (mmol/L)	0.62±0.10	0.73±0.06
Cholesterol (mmol/L)	1.70±0.40	2.50±0.79
Non-esterified fatty acids (mmol/L)	0.22±0.04	0.32±0.09

* *p*-value≤0.05; ** *p*-value≤0.01

**Figure 2:**
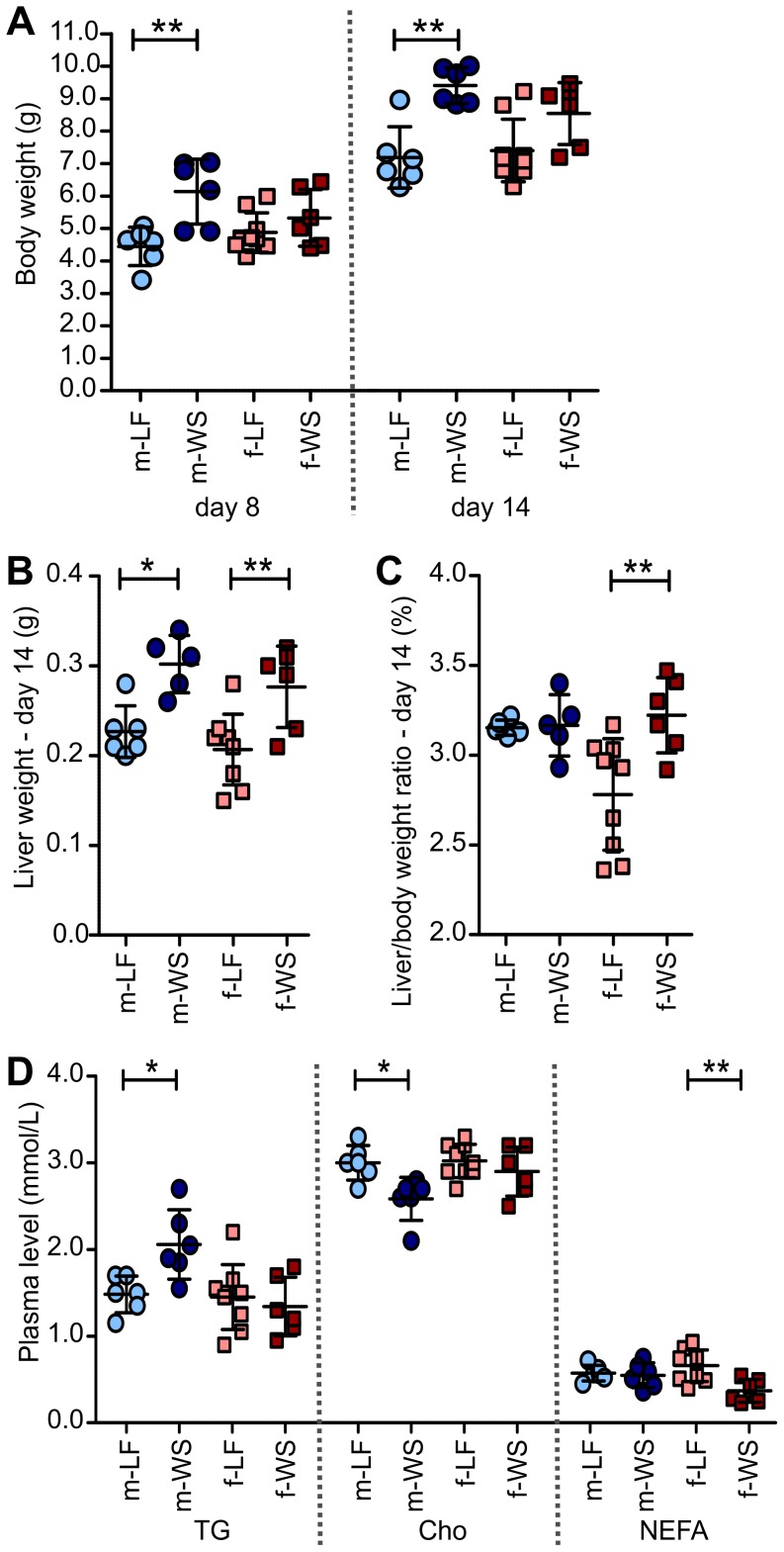
Offspring’s body, liver, and blood parameters. (A) Body weight at postnatal day (PD) 8 and 14, (B) liver weight at PD 14, (C) liver weight/body weight ratio, and (D) plasma triglycerides (TG), plasma cholesterol (Cho), and plasma non-esterified fatty acids (NEFA) were determined in offspring of maternal LFD or maternal WSD. Light blue circle = male/maternal LFD; dark blue circle = male/maternal WSD; pink square = female/maternal LFD; red square = female/maternal WSD. *p≤0.05; **p≤0.01.

The body weight of offspring from WSD and LFD dams at postnatal day (PD) 8 and PD14 (sacrifice) showed a significant increase over time that was independent from sex or diet (Figure 2A). At both time points, male offspring of WSD dams were significantly heavier than offspring of LFD dams. For female offspring, significant differences in body weight were not detected at any time (Figure 2A). In both sexes, liver weight was significantly higher at sacrifice in offspring of WSD dams, leading to a significant increased liver weight/body weight ratio in WSD females only (Figure 2B and C). For blood parameters, likewise differences between the sexes were observed: in male offspring of WSD dams, plasma TG and plasma cholesterol levels were significantly higher and lower, respectively, when compared with offspring from LFD dams (Figure 2D). In females, only NEFA plasma levels were affected by the mother’s diet, and were significantly lower in WSD offspring (Figure 2D).

### Changes in gene expression upon maternal WSD

**Figure 3:**
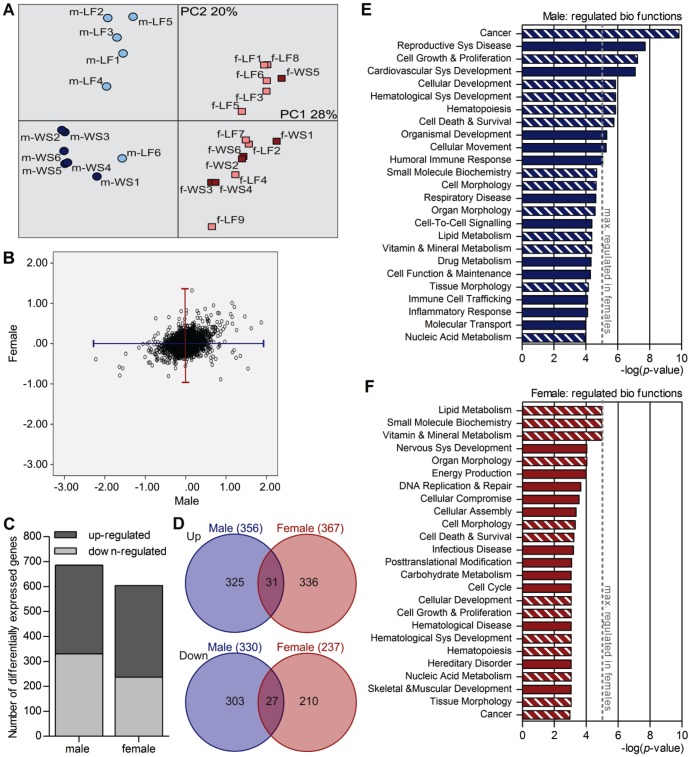
Maternal diet-induced profiles of liver gene expression and biological functions. (A) PCA was performed with the top 1,000 most variable genes (based on IQR) of the microarray analysis. (B) The gene expression response (displayed as 2log-ratio of maternal WSD-maternal LFD) of male and female offspring is plotted against each other. (C) By maternal WSD significantly up- or down-regulated genes (*p*≤0.01) are shown for male and female offspring. (D) Significantly up- or down-regulated genes (*p*≤0.01) in both sexes were compared and are shown as Venn diagrams. (E) and (F) Ingenuity Pathway Analysis (IPA) was carried out on genes that were significantly differentially expressed upon maternal WSD. The top 25 regulated biological functions in males and females respectively, are displayed. Hatched bars = biological functions that occur in the top 25 of both sexes. Filled bars = biological functions that are specific for one sex. Sys = System.

To evaluate the molecular effects of maternal WSD during the pre- and gestational period and during lactation, we performed microarray analysis (MA) on liver RNA from two-week-old offspring. The distribution of IBMT *p*-values of resulting data indicated a similar gene expression profile for males and females at baseline (maternal LFD) and differential gene expression between maternal WSD and LFD exposures in all offspring (data not shown). Principal component analysis (PCA) of the top 1,000 most variable genes was performed, using the inter quartile range (IQR) of all samples as indicator of variability of gene expression. As shown in Figure 3A, the samples distinctly separate into two clusters by principle component (PC) 1, which accounts for 28% of the gene expression variation. One cluster contains the male samples and the other one the female samples. Within the males, another moderate clustering by PC2 (20%) was detected. It mainly separates the group of male offspring from LFD dams from the group of male offspring from WSD. Still, one male pup of maternal LFD (m-LF6) rather clustered with the male offspring of the WSD dams. This is probably caused by an idiosyncratic gene expression profile, as this mouse showed similar behaviour in the heat maps (see below) of Wnt/beta-catenin pathway and fatty acid metabolism, but not in the cholesterol metabolism. In females, a pronounced second clustering by PC2 was not detectable. In addition, no clear separation was visible with further components (e.g. PC3 and PC4, data not shown).

Using the offspring of LFD dams as a reference, we calculated 2log ratios of gene expression in male and female offspring of WSD dams separately, to identify differentially expressed genes between diets within the two sexes. Thereby, a 2log ratio of 1 corresponds to a fold change (FC) of 2. Plotting the ratios for the whole gene set in a scatter plot (Figure 3B) revealed that gene expression of male offspring was strongly affected by maternal WSD, with ratios from −2.22 to 1.87. In females, the response was weaker (ratios between −0.89 and 1.32) and did not correlate with the male response. Although numerous genes in females were regulated in the same direction as in males upon maternal WSD, a substantial number was regulated oppositely.

In total, a maternal WSD caused approximately the same number of significantly (*p*≤0.01) differentially expressed genes in male and female offspring, 686 and 604 genes respectively (Figure 3C; for detailed lists of the differentially regulated genes and their functions indicated by the Gene Ontology Database see Gene lists S1). In both cases, about half of these differentially expressed genes were up-regulated and half were down-regulated by maternal WSD. Interestingly, comparison of the subsets of significantly up- and down-regulated genes revealed that only about 10% of the up- or down-regulated genes overlapped in both sexes (Figure 3D). In addition, there were also four genes that were significantly differentially expressed by maternal WSD, but in opposite directions in males and females (Table 2).

**Table 2:** Genes that are differentially regulated (*p*≤0.01) upon maternal WSD in male and female offspring (for functional information see Gene lists S1).

up-regulated in males and females		down-regulated in males and females		oppositely regulated
Akr1c13	Ncl	Ang	Prpf4	9030625A04Rik
Atp5c1	Nid2	Bmyc	Rarres1	Abcg8
Atp6v0e	Pcyt1a	Casp8	Rnase4	Mrgpre
Bcas2	Pgrmc2	Cldn25	Rnd2	Paqr9
Ccbl1	Phpt1	Gdf10	Sntb1	
Ces1e	Ppa1	Gfra1	Steap2	
Ces1g	Ppap2c	Grtp1	Syt1	
Chst3	Psmb10	Igsf9	Tmem200b	
Clec2h	Qdpr	Isyna1	Tox	
Ctnnbip1	Sdhb	Itih5	Zfhx2	
Dnahc11	Slc36a1	Leprotl1	Zfp27	
Idh2	Slc9a3r1	Mustn1		
Il7	Sqrdl	Mxra8		
Krt20	Susd1	Myo15b		
Meg3	Tead1	NA		
Mgea5		Olfml1		

### Changes in biological functions and pathways upon maternal WSD

To investigate which biological functions and pathways the differentially regulated genes affect, data were analysed using Ingenuity Pathway Analysis (IPA). As can be seen in Figure 3E and 3F, 13 of the top 25 regulated biological functions were regulated in both sexes, but differed in ranking. The remaining 12 regulated biological functions differed between males and females (Figure 3E and F). In IPA, the significance of regulation of a biological function is indicated by -log(*p*-values), which are calculated based on the number of regulated genes in relation to the total number of genes attributed to the function. Thereby, a -log(*p*-value) of 10 relates to a *p*-value of 1.0*10^−10^ and a -log(*p*-value) of 5 relates to a *p*-value of 1.0*10^−5^. In males, the top 10 regulated biological functions had -log(*p*-values) between 9.82 and 5.28. In females, the regulation of biological functions was less pronounced, with maximal -log(*p*-value) of 4.96.

In males, the strongest regulated biological functions included most notably (cell) developmental functions, such as *Cellular Growth & Proliferation*, *Cellular Development*, and *Haematological System Development*. In contrast, in females, especially functions that relate to metabolism and energy homeostasis were among the highest regulated functions, including *Lipid metabolism*, *Vitamin & Mineral Metabolism*, and *Energy Production*. Except for *Energy Production*, the above-mentioned functions were also listed in the top 25 of the other sex, but on a much lower rank.

### Differential gene expression of the Wnt2-related gene set

**Figure 4:**
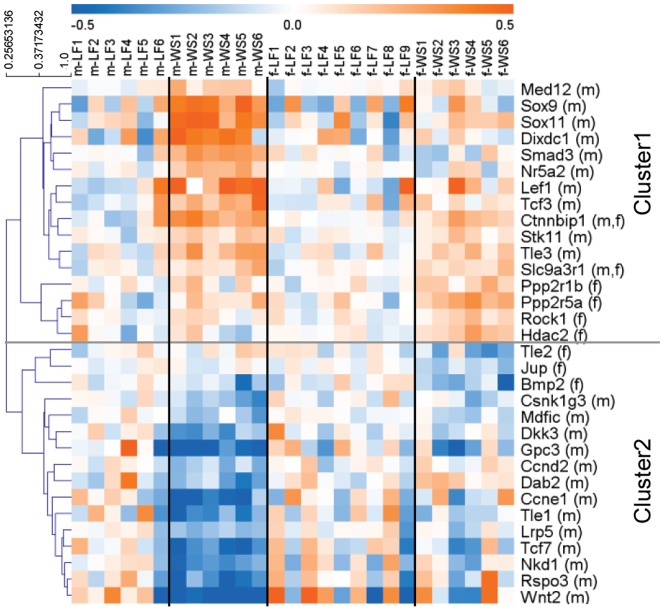
Heat map of Wnt/beta-catenin pathway. The Wnt/beta-catenin gene set was hierarchically clustered based on Pearson correlation. Gene expression values are displayed on colour scale: blue indicates lower values than average of maternal LFD-group of respective sex; orange indicates higher values than average of maternal LFD-group of respective sex. (m) = gene was significantly changed in male offspring upon maternal WSD. (f) = gene was significantly changed in female offspring upon maternal WSD. *p*-values≤0.01 were considered significant.

**Figure 5:**
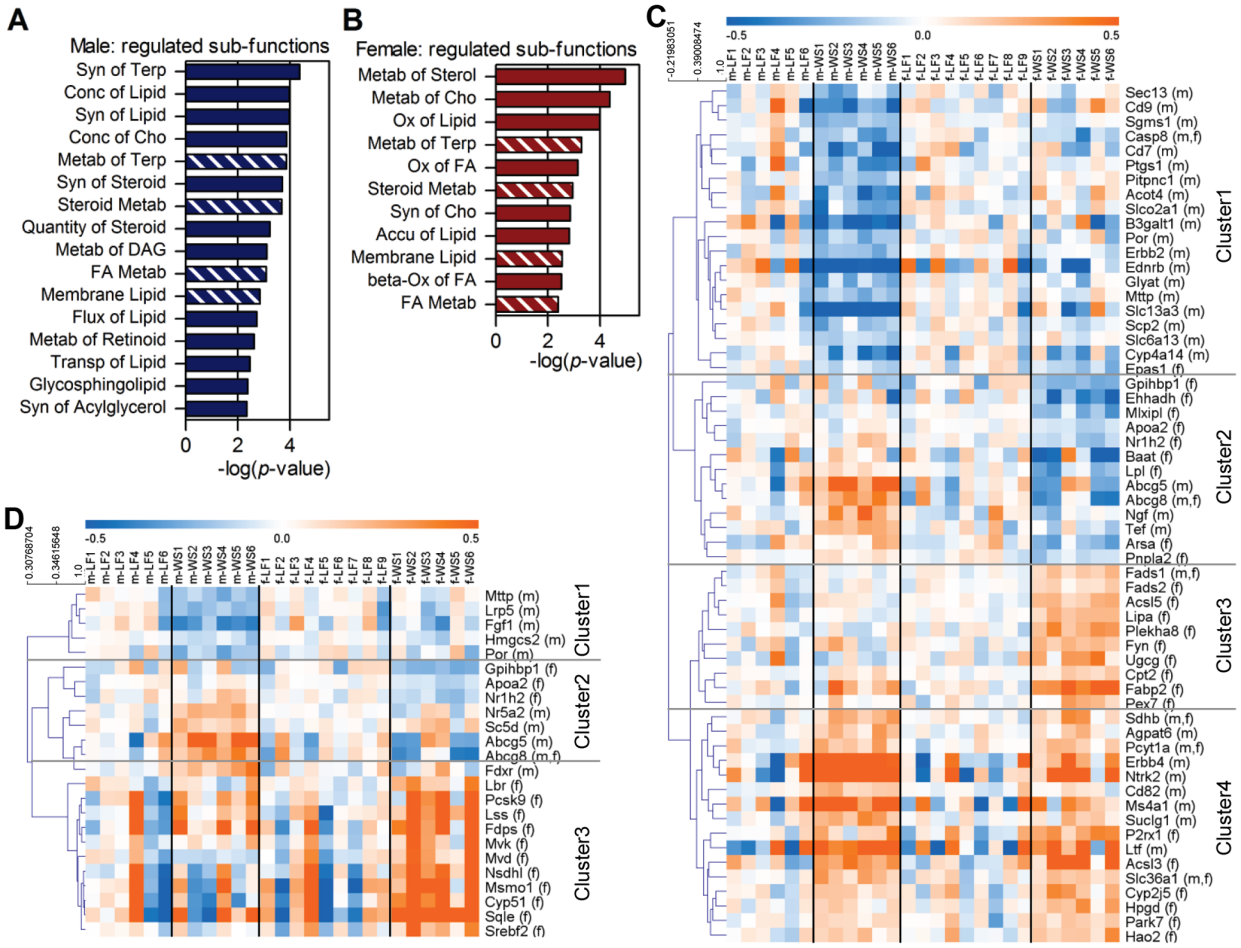
Lipid metabolism in offspring of maternal WSD represented by sub-functions and heat maps. (A) and (B) display sub-functions of lipid-metabolism that were according to IPA significantly regulated (*p*≤0.05) upon maternal WSD in males or females respectively and contained≥7 regulated molecules. Hatched bars = sub-functions that are regulated in both sexes. Filled bars = sub-functions that are specific for the sex. Syn = Synthesis; Conc = Concentration; Metab = Metabolism; Transp = Transport; Accu = Accumulation; Terp = Terpenoid; Cho = Cholesterol; DAG = Diacylglycerol; FA = Fatty Acid. (C) and (D) show hierarchical clustered heat maps of the fatty acid metabolism gene set and the cholesterol metabolism gene set, respectively. Hierarchical clustering of gene sets was based on Pearson correlation. Gene expression values are displayed on colour scale: blue indicates lower values than average of maternal LFD-group of respective sex; orange indicates higher values than average of maternal LFD-group of respective sex. (m) = gene was significantly changed in male offspring upon maternal WSD. (f) = gene was significantly changed in female offspring upon maternal WSD. *p*-values≤0.01 were considered significant.

Various pathways and sub-functions correspond to biological function categories in IPA. Figure 4 and 5 display heat maps of gene sets that relate either to developmental or metabolic functions, which were regulated upon maternal WSD according to IPA. Represented gene sets were received from the integrated knowledge base of IPA and the KEGG pathway database. Only genes significantly differing in gene expression depending on maternal diet in at least one of the sexes are displayed.

The Wnt/beta-catenin signalling pathway (Figure 4), which is a representative pathway related to cell development, growth, and proliferation, was indicated by IPA to be significantly regulated in males upon maternal WSD. The responding gene set contained 32 genes, which showed moderate interindividual variability of gene expression at baseline (maternal LFD). Basal expression levels of the Wnt/beta-catenin pathway genes were alike in both sexes, with exception of two genes (*Smad3* and *Tle2*), which were significantly but only slightly differentially expressed in male and female offspring of maternal LFD (FC = 1.13 and 1.14 respectively).

Upon exposure to maternal WSD, clear and interindividual steady differential expression of 25 genes of the Wnt/beta-catenin set was detected in male offspring. By hierarchical clustering of the genes based on Pearson correlation, two main clusters were formed, largely representing subsets of up- and down-regulated genes in male offspring from maternal WSD. The subset of up-regulated genes primarily contained factors that are indicated as negative regulators of Wnt/beta-catenin signalling (*Med12*, *Sox9*, *TCF3*, *Ctnnbip1*, *Hdac2*), or as negative regulators of cell proliferation (*Stk11*, *Slc9a3r1*). The set of down-regulated genes predominantly consisted of factors indicated as direct members of the Wnt/beta-catenin signalling pathway (*Wnt2*, *LRP5*, *TCF7*), or as positive regulators of Wnt/beta-catenin signalling (*Bmp2*, *Dab2*). There were few exceptions to these findings within the clusters: *DKK3* and *Gpc3*, which are negative regulators of Wnt/beta-catenin signalling, were among the down-regulated genes, and *Dixdc1* and *SMAD3*, which are indicated as positive regulators were among the up-regulated genes.

In female offspring, the overall effects of maternal WSD were interindividually inconsistent and more subtle than in males. Only nine genes were significantly affected, forming a small subgroup of mostly up-regulated genes. Even though the group contained the two genes that were significantly regulated in both sexes (*Ctnnbip1* and *Slc9a3r1*), the sets of up-regulated genes in males and females differed essentially from each other. A set of consistently down-regulated genes in females was not clearly distinguishable.

### Different sub-functions and differential gene expression of the lipid metabolism gene sets

For males, IPA indicated 16 different sub-functions of lipid metabolism that were significantly regulated (*p*≤0.05) and contained at least seven molecules (Figure 5A). In females, 11 sub-functions of lipid metabolism matched the same criteria (Figure 5B). Yet, only four of these sub-functions overlapped in males and females.

For selected sub-functions of lipid metabolism - *Fatty Acid Metabolism, Metabolism of Cholesterol,* and *Concentration of Cholesterol* - the relevant gene lists were extracted from IPA and heat maps were generated. According to IPA, *Fatty Acid Metabolism* (represented by a gene set of 58 genes) was regulated in both sexes. Basal ( =  maternal LFD) expression levels of all genes were similar in males and females, showing moderate interindividual variability (Figure 5C). In offspring from WSD dams, pronounced differential gene expression was seen in both sexes. By hierarchical clustering based on Pearson correlation, four main clusters were formed (uppermost = cluster 1; lowermost = cluster 4). Cluster 1 and cluster 4 represented the groups of down- and up-regulated genes in male offspring, respectively. In females, cluster 2 containing the down-regulated genes and cluster 3 and 4 containing the up-regulated genes of *Fatty Acid Metabolism*. Thereby, five genes of the whole set were significantly regulated in both sexes (*Abcg8*, *Casp8*, *Pcyt1a*, *Sdhb*, *Slc36a1*), and 53 genes only in one sex.

*Cholesterol Metabolism* and *Cholesterol Concentration* were indicated to be significantly regulated exclusively in female and male offspring, respectively. The combined gene set consisted of 24 genes, whose basal expression levels were similar in both sexes, but showed moderate to strong interindividual variation (Figure 5D). Upon maternal WSD, differential gene expression in both sexes was visible. The three clusters (cluster 1–3) that formed upon hierarchical clustering with Pearson correlation contained either down-regulated genes (cluster 1) or up-regulated genes (cluster 2) in males, or up-regulated genes (cluster 3) in females. The up-regulated genes in males included factors relevant for cholesterol transport (*Abcg5*, *Abcg8*, *Apoa2*, *Nr1h2*), while the up-regulated genes in females predominantly related to cholesterol biosynthesis. Only one gene (*Abcg8*) was significantly regulated in both sexes.

In summary, the data presented in Figure 5 show that gene expression at baseline did not differ between the sexes, but interindividual variations were observed. Upon exposure to maternal WSD pronounced gene expression changes occurred in males and females that differed clearly between the sexes. Furthermore, depending on the biological function certain individual mice had stronger responses than others, but not always the same individual mice had the strongest response.

## Discussion

To date, the concept of metabolic programming, which refers to the link of maternal nutrition with the predisposition of the offspring for non-communicable diseases, is widely accepted. Rodent studies consistently demonstrate that maternal high fat intake is associated with the development of metabolic abnormalities in adolescent and adult offspring. In general, this is independent from the sex and the post-weaning diet of the offspring [26], [38], [39]. However, the specific nature and severity of the observed abnormalities, including obesity, disturbed glycaemic control, and fatty liver, can still be influenced by sex and the post-weaning diet, respectively [23], [24], [27]. A likely explanation for this may be that similar outcomes (fatty liver, body weight changes, etc.) can putatively be achieved by different mechanisms in males and females. Therefore, on top of the physiology the underlying molecular networks need to be characterised. Moreover, it is critical to study the initiating phase of programming, thus when the offspring is still under the influence of the maternal diet.

While a large number of publications deal with the long-term consequences of varying maternal diets on the offspring’s physiology and health in adulthood, the immediate effects in young offspring are mostly unexplored. To get more insight into this early phase, in the present study, we analysed young offspring at a pre-weaning time point, eliminating most influencing factors other than maternal diet. Apart from determining physiological parameters, we also monitored the liver transcriptome in male and female offspring. This approach allowed us to (i) characterise the molecular impact of maternal WSD and its relation to the physiological outcome, (ii) to illuminate sex-specific effects, and (iii) to identify molecular aspects that went unnoticed to date by the usual function-focussed strategies. So, here, we observed that young mouse offspring of maternal WSD showed changes in physiological parameters, including significantly higher body weight and liver weight, as well as altered plasma lipids. The amplitude of these changes depended on the sex of the offspring.

To identify underlying direct effects of maternal diet on the offspring that relate to the observed physiological differences and on possibly adverse health effects in later life, we analysed the hepatic molecular status of the young mice in a universal approach and linked it to biological functionality. Within our transcriptome-wide analysis of the offspring’s livers, we detected that the gene expression profiles upon maternal LFD were similar in males and females, but maternal WSD induced clear differences in the expression profiles between the sexes. This demonstrates that on transcriptional level males and females react differently upon maternal WSD. Within both sexes, gene expression profiles of the maternal diet groups were different from each other, with a more pronounced maternal diet effect in males indicated by the distinct clustering of diet groups in the PCA. In females, however, further factors seem to be involved in the manifestation of variability, which does not exclude that, compared to males, a more subtle effect of the diet on the transcriptome is present in the females.

Correlating gene expression changes in males and females showed that male offspring had in general a more pronounced transcriptome-wide response to maternal diet. Furthermore, in some cases, the responses were opposite in both sexes. Although the number of significantly regulated genes (*p*≤0.01) was similar in both males and females, they overlapped only slightly. Overall, this indicates that the molecular status of young offspring of both sexes is in general affected by maternal WSD, but that sex-differences occur regarding strength and nature of the response. This is in accordance with earlier findings of sex-dependent molecular effects in adult offspring [21]–[28], [40], and extends them to a global level.

Applying IPA to explore the biological significance of the molecular impact of maternal WSD, we found that the relatively small overlap of regulated genes in both sexes translates into a similar result for the potential biological functionality of the regulated genes: only half of the top 25 regulated functions, identified by IPA, overlapped in males and females, and they ranked completely different. A similar observation was made in a maternal high-fat diet study by Gabory et al. for sex-specificly regulated genes and indicated biological function in the mouse placenta [41]. Interestingly, consistent with our findings that males respond stronger on a molecular level to the challenge by maternal diet, they also showed stronger reactions on a functional level, as indicated by the high significance of regulation of biological functions, expressed as –log(*p*-value). Thus, males and females seem to have a specific prioritisation of which biological functions are regulated with a certain effort upon stimuli exposure.

According to IPA, in liver from male offspring, predominantly regulation of developmental functions was induced by maternal WSD. One pathway that is highly associated with developmental functions is the Wnt/beta-catenin pathway. No sex-specific differences were detected for the expression levels of Wnt/beta-catenin pathway genes at baseline. However, upon maternal WSD, pronounced differential expression was observed in male offspring only, emphasizing that maternal WSD affects the two sexes differently. On the whole, we detected for the male offspring from maternal WSD that direct Wnt/beta-catenin pathway genes and positive regulators of Wnt/beta-catenin signalling were down-regulated, and negative regulators of Wnt/beta-catenin signalling and cell proliferation were up-regulated.

In general, Wnt/beta-catenin signalling is crucial for embryonic development and homeostatic self-renewal in adulthood [42]. When Wnt binds to its receptor, beta-catenin is stabilised and translocates to the nucleus. There, it induces the transcription of the beta-catenin target gene battery leading to cell proliferation and the direction of cell fate [43]–[45]. For liver in particular, this process not only controls development, growth, and regeneration, but also regulates hepatic zonation, haematopoiesis, and various metabolic functions, including energy substrate metabolism and detoxification processes [46]–[49]. Disruption of proper Wnt/beta-catenin signalling during development, such as conditional beta-catenin knockout, leads to decreased liver growth, diminished hepatocyte proliferation, and sustained haematopoiesis, indicating delayed liver development [50].

Overall, our results imply that the Wnt/beta-catenin pathway is inhibited in male offspring of maternal WSD at two weeks postnatally. Since proper beta-catenin activity is limited to certain developmental periods, including embryonic day 10 to 14 and postnatal day 5 to 20 [50], [51], the decrease of Wnt/beta-catenin activity at this time point could imply impaired liver development [50]. However, that would be in contrast with the increased liver weight of male offspring of maternal WSD that we detected in our study, despite hepatocyte specific proliferation markers showing no differential expression. In addition, plasma cholesterol was decreased in the relevant male pups. Thus, decreased Wnt/beta-catenin signalling by maternal WSD might affect cholesterol homeostasis in males, causing the observed increased liver weight by lipid accumulation in the liver, next to steady or impaired hepatocyte proliferation. This theory should be verified by physiological liver data in future studies, but it is supported by Behari *et al.*, who associated inhibited beta-catenin signalling with defective cholesterol homeostasis and increased hepatic cholesterol accumulation and steatosis [52].

As mentioned above, lipid metabolism is one of the most extensively discussed issues with respect to maternal HFD, but only a limited number of studies considered sex differences [23], [27]. The present study demonstrates for the first time that regulation of lipid metabolism by maternal WSD occurs already in young, pre-pubertal mice in a sex-specific manner. This includes that different sub-functions of lipid metabolism were affected in both sexes, as indicated by IPA, and that also the sub-functions themselves were regulated sex-dependently, which is shown convincingly by the heat maps, zooming in on fatty acid and cholesterol metabolism. For cholesterol metabolism, the sex-specific effects of maternal WSD are especially remarkable: in males, primarily genes related to cholesterol homeostasis were regulated. This is in accordance with the findings that decreased Wnt/beta-catenin signalling might result in defective cholesterol homeostasis [52], hence could contribute to the changes in liver weight and plasma lipids observed in males, as indicated above. In contrast, in females, genes of the cholesterol biosynthesis were predominantly up-regulated, suggesting an increased synthesis of cholesterol. Since plasma cholesterol levels did not change in females of maternal WSD, but liver weight increased significantly, it is conceivable that accumulation of cholesterol in the liver might have contributed to the increased liver weight, which should be tested in future studies. Overall, it seems that the equal physiological phenotypes of increased liver weight in male in female offspring might result from different sex-specifically regulated mechanisms.

Sexual dimorphism of lipid and cholesterol metabolism is described in humans and animal models, especially in relation to cardiovascular diseases and cholelithiasis [53], [54]. How the dimorphism develops is not well understood, but sex-specific regulation and activation of relevant transcription factors by (sex) hormones is indicated [55]–[57]. Circulating sex hormone levels in the early postnatal phase are not well fathomed, but they are expected to be similarly low in male and female young mice [58]. Hence, the direct effect of sex hormones in our study is questionable, but could be considered in future research. Apart from this, brain sexual dimorphism might be relevant for the observed sex-specific effects. Brain sexual differentiation starts *in utero* and concerns – apart from the usually discussed reproduction-related circuits – many brain regions, including the *nucleus arcuatus*, which is crucial for energy homeostasis [59], [60]. Hence, central metabolism regulation might already be affected at this young age in a sex-dependent manner. Related to this, different metabolic load and metabolic plasticity in both sexes might contribute to the differences in male and female offspring. Thus, further parameters like suckling amounts of different individuals should be analysed in future research.

## Conclusion

In conclusion, effects of maternal WSD can already be observed in two-week-old offspring in the present model. Except for the liver weight, we observed pronounced differences between male and female offspring for all measured parameters, including the liver transcriptomes. The cause of this sex-specificity is unclear and requires further research.

Furthermore, on the background of the metabolic programming concept, it has to be explored how persistent the effects observed in young offspring are, and how they relate to the molecular and health status of adult offspring. Herein lies the potential to identify early markers of adverse health effects in later life that could be used to develop enhanced early diagnosis, prevention, and treatment strategies of non-communicable diseases, such as the metabolic syndrome.

## Supporting Information

Figure S1 **Confirmation of microarray results by quantitative real-time PCR.** To validate the microarray data, we analysed the expression of various genes by quantitative real-time PCR. Displayed is a selection of 8 genes, which were in the microarray either significantly regulated in males (Ces3b, Cyp2b10, Wnt2), in females (Cyp51), in both sexes (Abcg8, Akr1c13), or not at all (Lxra, Ppara). For each gene, the left panel (A1–H1) displays the microarray results (signal intensity), and the right panel (A2–H2) displays the corresponding quantitative real-time PCR (qPCR) measurements. For the qPCR analyses, the mRNA levels were standardized to the reference gene 36B4 (relative expression). Horizontal bars and whiskers represent mean values ± SD. Significance was determined by intensity based-moderated t-statistics implementing empirical Bayes correction (microarray data) or unpaired student’s t-test (qPCR data). *p≤0.05. MA = microarray. Light blue circle = male/maternal LFD; dark blue circle = male/maternal WSD; pink square = female/maternal LFD; red square = female/maternal WSD.
(TIF)

Method S1 **Quantitative real-time PCR, including primer sequences.**
(PDF)

Gene lists S1 **Detailed lists of the differentially regulated genes in both sexes, including the indicated gene functions (based on Gene Ontology Database).**
(XLSX)
