# Supplementary material for: Maternal Western-Style High Fat Diet Induces Sex-Specific Physiological and Molecular Changes in Two-Week-Old Mouse Offspring
Source: PLoS One. 2013 Nov 5;8(11):e78623. doi: 10.1371/journal.pone.0078623 (PMC3818485; doi:10.1371/journal.pone.0078623)
Supplement: Method S1 — Quantitative real-time PCR, including primer sequences. (PDF) [file pone.0078623.s002.pdf]

Method S1: Quantitative real-time PCR .

To validate the microarray data for all offspring samples, each 1 µg purified liver RNA was used to synthesize single-stranded complementary DNA (cDNA). Therefore, the “First Strand cDNA synthesis Kit” (Fermentas, Thermo Scientific) was used following the supplier's protocol for random hexamer primers. Resulting cDNA was stored at -20°C until further use. Quantitative real-time PCRs of the cDNA (2 µl/sample) were performed using the “SensiMix SYBR No-ROX Kit” (Bioline) and gene specific primers obtained from Eurogentec (Seraing, Belgium; see Table 3 below). Primer sequences were retrieved from the online PrimerBank database [1], or otherwise designed using the Primer3 program [2]. Primers were tested for specificity by BLAST analysis. The following thermal cycling conditions were applied in a C1000 Thermal Cycler, CFX 384 Real-Time System cycler (Bio-Rad laboratories BV, Veenendaal, The Netherlands): denaturation for 2 minutes at 94°C, followed by 40 cycles of 15 seconds denaturation at 94°C, and 45 seconds annealing and elongation at 60°C. PCR reactions were performed in duplicate, and all samples values were calculated by using a standard curve and standardized to the reference gene 36B4. The reference gene was selected after testing different reference genes (36B4, 18S), which led to identical results.

Table S1: Sequences of primers used for quantitative real-time PCR analysis.

Gene	Forward	Reverse
Abcg8	5’-GACAGCTTCACAGCCCACAA-3’	5’-GCCTGAAGATGTCAGAGCGA-3’
Akr1c13	5’-CTGGAGCGAATCCTCAATAAGC-3’	5’-GGGGAGTTTTGGTCCACCC-3’
Ces3b	5’-GGATTGTCACGACCATTATGATG-3’	5’-CAGAGCCACAGGCCACAGAA-3’
Cyp2b10	5’-AAAGTCCCGTGGCAACTTCC-3’	5’-TTGGCTCAACGACAGCAACT-3’
Cyp51	5’-CTGAGAAGCTCTCGTGCTGT-3’	5’-TTCAAATGCCATTCGGTCT-3’
Lxra	5’-GCTCTGCTCATTGCCATCAG-3’	5’-TGTTGCAGCCTCTCTACTTGGA-3’
Ppara	5’-TATTCGGCTGAAGCTGGTGTAC-3’	5’-CTGGCATTTGTTCCGGTTCT-3’
Wnt2	5’-CATAGCCCCCCACCACTGT-3’	5’-AGTTCCTTCGCTATGTGATGTTTCT-3’
36B4	5’-AGCGCGTCCTGGCATTGTCTGTGG-3’	5’-GGGCAGCAGTGGTGGCAGCAGC-3’

1. Wang X, Seed B (2003) A PCR primer bank for quantitative gene expression analysis. Nucleic Acids Res 31: e154.
2. Rozen S, Skaletsky H (2000) Primer3 on the WWW for general users and for biologist programmers. Methods Mol Biol 132: 365-386.
